# Multimodal Imaging in Rhinoorbitocerebral Mucormycosis Associated with Type 2 Diabetes After COVID-19

**DOI:** 10.5041/RMMJ.10483

**Published:** 2022-10-27

**Authors:** Pavel Mikhailovich Zelter, Olesya Vladimirovna Zeleva, Egor Andreevich Sidorov, Dmitriy Vyacheslavovich Solovov, Evgeniy Nikolaevich Surovtsev

**Affiliations:** 1 Radiology Department, Clinic of Samara State Medical University, Samara, Russia; 2 Department of Radiology, Samara State Medical University, Samara, Russia; 3 Otorhinolaryngology Department, Clinic of Samara State Medical University, Samara, Russia

**Keywords:** Computed tomography, coronavirus infection, COVID-19, magnetic resonance imaging, maxillary sinuses, mucormycosis, paranasal sinus

## Abstract

**Purpose:**

This case series analyzed the appropriateness of computed tomography (CT) and magnetic resonance imaging (MRI) for visualization of rhinoorbitocerebral mucormycosis (ROCM) patterns associated with type 2 diabetes (T2D) post-recovery from coronavirus disease 2019 (COVID-19).

**Methods:**

The study included 24 patients with invasive ROCM after having recovered from COVID-19. All patients underwent CT examinations and microbiological and histological verification; 5 patients underwent MRI.

**Results:**

The CT and MRI patterns noted in our patients revealed involvement of skull orbits, paranasal sinuses, large arteries, and optic nerves, with intracranial spread and involvement of the cranial base bones. Using brain scan protocol for CT provided better soft-tissue resolution. We found that extending the MRI protocol by T2-sequence with fat suppression or STIR was better for periantral fat and muscle evaluations.

**Conclusion:**

Computed tomography of the paranasal sinuses is the method of choice for suspected fungal infections, particularly mucormycosis. However, MRI is recommended if there is suspicion of orbital, vascular, or intracranial complications, including cavernous sinus extension. The combination of both CT and MRI enables determination of soft tissue invasion and bony destruction, thereby facilitating the choice of an optimal ROCM treatment strategy. Invasive fungal infections are extremely rare in Europe; most of the related data are provided from India and Middle Eastern or African nations. Hence, this study is notable in its use of only diagnosed ROCM cases in Russia.

## INTRODUCTION

Rhinoorbitocerebral mucormycosis (ROCM) is an aggressive fungal infection that is potentially lethal. The infection involves the sinuses but may destroy facial bones and invade other skull structures. It commonly occurs in immunocompromised patients following hormonal, monoclonal antibody, or broad-spectrum antibiotics therapies. One risk factor stands out, namely uncontrolled diabetes mellitus.

Before the coronavirus disease 2019 (COVID-19) pandemic, mucormycosis was reported in isolated cases from different places in the world. Most of these patients had a background of oncological or hematological disease. However, since the onset of the pandemic, more patients have been reported with ROCM after having been treated for COVID-19.^1^

A recent publication by the European Confederation of Medical Mycology and the International Society of Human and Animal Mycoses identified 80 cases of COVID-19-associated ROCM.^2^ The median age of these patients was 55 years (range 10–86 years); 79% were males. Overall mortality was 31%. Similar results were published by Singh et al.^3^

The aggressive nature of ROCM requires immediate diagnosis and treatment.^4^ Hence, empirical antifungal therapy should be initiated in patients with clinical signs of ROCM, before confirming the diagnosis by microbiological or histopathological methods.^5^ Diagnostic imaging is vital in supporting the diagnosis and visualization of the extent of involvement of the orbital structures and brain.

Mucormycosis spreads by invading the adjacent tissues, causing bony destruction, and spreading through the bone and anatomical channels (nasolacrimal ducts, lymphatic vessels, and neurovascular bundles).^6^ Comprehensive assessment of mucormycoses requires computed tomography (CT) or magnetic resonance imaging (MRI), particularly aimed at imaging these anatomical regions. Mazzai et al. published a pictorial review of mucormycosis from onset to vascular complications, and proposed a three-stage grading system of anatomical structure involvement (Table 1),^8^ which was expanded upon by Kumar et al.^9^ The three stages include sinonasal, orbital, and intracranial involvement. The objective of this study was to describe CT and MRI findings in 24 patients with ROCM associated with type 2 diabetes (T2D) and COVID-19 infection.

**Table 1:** Proposed Three-Tier Staging of Rhinoorbitocerebral Mucormycosis.^7^

Stage	Details
Stage 1: Sinonasal Involvement	1A: Involvement of 1 sinus and ipsilateral middle turbinate
	1B: Involvement of >1 ipsilateral sinus and/or turbinate
	1C: Involvement of bilateral sinonasal cavities
Stage 2: Orbital Involvement	2A: Involvement of medial and/or inferior orbital compartment only
	2B: Diffuse unilateral orbital involvement with or without optic nerve, nasolacrimal duct, and vascular involvement
	2C: Bilateral orbital involvement
Stage 3: Central Nervous System Involvement	3A: Involvement of pachymeninges, cribriform plate, cavernous sinus/Meckel’s cave
	3B: Vascular involvement (infarct/bleed)/perineural spread and skull base involvement
	3C: Leptomeningitis, cerebritis or abscess formation—focal or diffuse involvement

## METHODS

**Figure 1:**
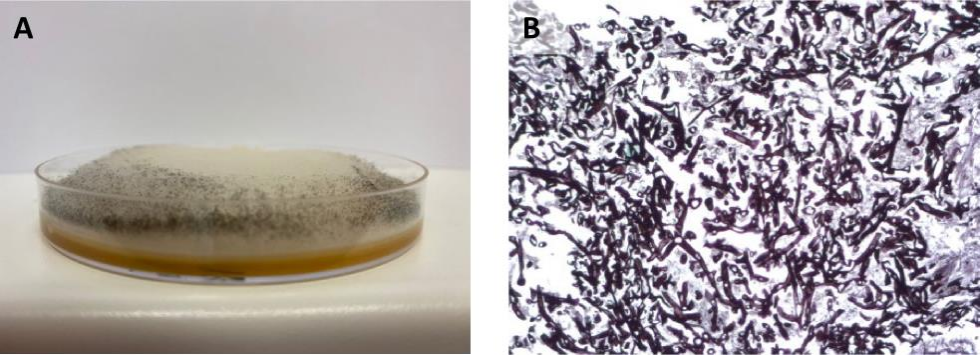
Histological Examination for Mucormycosis. **A:** Mucormycosis cultured from tissue samples. **B:** Microscopic view of maxillar content (400×, silver impregnation). Note the black zygomycete hyphae.

This case series study was approved by the local ethics committee (approval ID: 12-2021). The study included 24 diabetic patients who had recovered from diagnosed COVID-19 and presented with ROCM complications. All patients were hospitalized in the ENT Department of Clinics of Samara State Medical University between November and December 2021. As part of their workup, the patients underwent chest and paranasal sinus CT scans via a 128-slice Revolution EVO CT scanner (General Electric, Moscow, Russia) with and without contrast enhancement; 5 of these patients also underwent contrast-enhanced MRI of the skull orbits and brain using the 1.5-T Magnetom Aera (Siemens AG, Munich, Germany). All patients underwent surgical treatment, and tissue samples were retrieved for histological examination for mucormycosis (Figure 1).

## RESULTS

**Figure 2:**
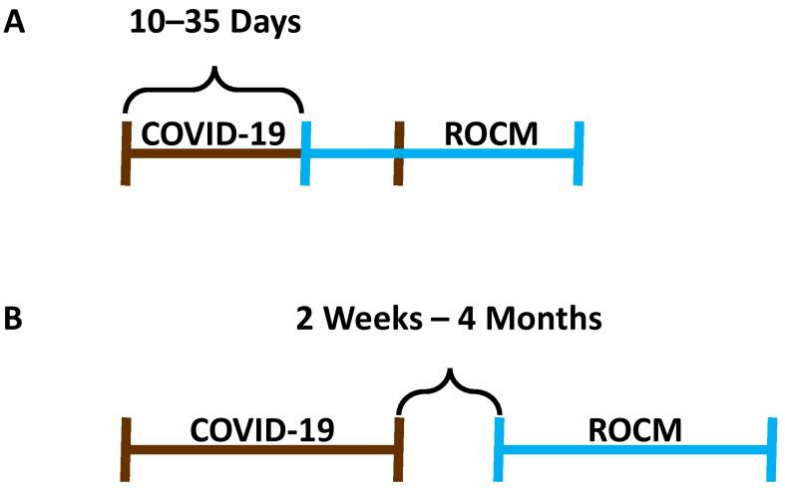
Development of Rhinoorbitocerebral Mucormycosis (ROCM) in Relation to Coronavirus Disease 2019 (COVID-19). **A:** Development of ROCM during COVID-19 hospitalization (*n*=7). **B:** Development of ROCM after hospital discharge for COVID-19 (*n*=17).

Twenty-four patients were included in this analysis: 13 females, 11 males; median age was 62 years (range 39–74 years). Upon COVID-19 diagnosis, all patients were treated with glucocorticoids, broad-spectrum antibiotics, immunomodulatory drugs, anticoagulants, and vitamins. The duration of COVID-19 hospital treatment ranged from 10 to 40 days. In 7 patients, ROCM developed between days 10 and 35 (mean 25 days) of their primary hospitalization for COVID-19. In 17 other patients, ROCM developed 2 weeks to 4 months (median 1.5 months) after hospital discharge and recovery from COVID-19. The development of ROCM in relation to COVID-19 is graphically presented in Figure 2.

All 24 patients suffered from T2D, 11 of which were first-time diagnoses while infected with COVID-19. Two patients were hospitalized for diabetic ketoacidosis coma with blood glucose levels up to 40 mmol/L (720 mg/dL). Common clinical symptoms of ROCM in our patients included headache, nasal breathing difficulty, nasal discharge (mucopurulent or bloody), facial soft tissue swelling, and visual impairment.

Chest CT scans in all patients revealed bilateral interstitial pneumonia in the form of reticular changes in the lung tissue with curvilinear sealing; no evidence of pulmonary fungal infection was identified in any of the CTs.

Paranasal sinus CT was performed in all 24 patients, 15 of which were done with contrast enhancement. Five patients also underwent contrast-enhanced cerebral and orbital MRI to better visualize ROCM invasion.

Table 2 summarizes the CT and MRI findings in all patients, including patient mortality.

**Table 2:** Computed Tomography and Magnetic Resonance Imaging Patterns in Patients and Mortality.

#	Patient Information (Age, Sex, Admission Details, Diabetes Status, Imaging)	Stage^8^	Perianthral Cellular Tissue Invasion	Bone Destruction	Orbit Involvement	Optic Nerve Invasion	Spread to Palate Fossa	Nasolacrimal Duct and Lacrimal Sac Invasion	Intracranial Structures Involvement	ROCM Mortality
1	62, F, admitted for ROCM S/Sx after COVID-19 discharge, T2D, CT + MRI	3C	+	+	+	+	+	−	+	−
2	39, M, admitted for ROCM S/Sx after COVID-19 discharge, T2D*, CT	2B	+	+	+	+	+	−	−	−
3	60, M, admitted for ROCM S/Sx after COVID-19 discharge, T2D*, CT	2A	+	+	+	−	−	−	−	+
4	42, F, admitted for ROCM S/Sx after COVID-19 discharge, T2D*, CT	2B	+	+	+	−	+	−	−	+
5	57, M, ROCM S/Sx while hospitalized with COVID-19, T2D*, CT	2A	+	+	+	−	+	−	−	−
6	58, M, admitted for ROCM S/Sx after COVID-19 discharge, T2D, CT	1B	+	+	−	−	+	−	−	−
7	48, F, admitted for ROCM S/Sx after COVID-19 discharge, T2D, CT	2B	+	+	−	−	+	+	−	−
8	69, M, admitted for ROCM S/Sx after COVID-19 discharge, T2D (diabetic coma), CT + MRI	3B	+	+	+	+	−	−	+	+
9	74, F, ROCM S/Sx while hospitalized with COVID-19, T2D*, CT	3A	−	+	+	−	−	−	−	−
10	67, F, admitted for ROCM S/Sx after COVID-19 discharge, T2D, CT	2A	−	+	+	−	−	−	+	-
11	73, F, admitted for ROCM S/Sx after COVID-19 discharge, T2D, CT	2A	−	−	+	+	−	−	−	−
12	56, M, admitted for ROCM S/Sx after COVID-19 discharge, T2D, CT	2B	−	+	−	−	−	+	−	−
13	69, M, ROCM S/Sx while hospitalized with COVID-19, T2D*, CT	1B	−	+	+	−	−	−	−	−
14	66, M, admitted for ROCM S/Sx after COVID-19 discharge, T2D (diabetic coma), CT + MRI	3A	+	+	+	+	−	−	+	−
15	49, F, ROCM S/Sx while hospitalized with COVID-19, T2D*, CT	2B	+	+	+	+	−	−	−	−
16	74, F, ROCM S/Sx while hospitalized with COVID-19, T2D, CT	2B	−	+	+	+	−	−	−	−
17	63, F, ROCM S/Sx while hospitalized with COVID-19, T2D*, CT	1B	+	+	−	−	−	−	−	−
18	62, M, admitted for ROCM S/Sx after COVID-19 discharge, T2D (diabetic coma), CT + MRI	3B	−	+	+	+	+	+	+	+
19	58, F, admitted for ROCM S/Sx after COVID-19 discharge, T2D, CT	2B	−	+	+	+	−	−	−	−
20	70, F, admitted for ROCM S/Sx after COVID-19 discharge, T2D (severe form), CT + MRI	3B	−	+	+	−	+	+	+	−
21	62, M, ROCM S/Sx while hospitalized with COVID-19, T2D*, CT	2B	−	+	+	+	−	−	−	−
22	68, M, admitted for ROCM S/Sx after COVID-19 discharge, T2D (severe form), CT	3A	+	+	+	+	−	−	+	+
23	55, F, admitted for ROCM S/Sx after COVID-19 discharge, T2D*, CT	1B	−	+	−	−	−	−	−	−
24	60, F, admitted for ROCM S/Sx after COVID-19 discharge, T2D*, CT	1A	−	+	−	−	−	−	−	−

* Type 2 diabetes (T2D) first identified during hospitalization with COVID-19.

+, present; −, not present; COVID-19, coronavirus disease 2019; CT, computed tomography; F, female; M, male; MRI, magnetic resonance imaging; ROCM, rhinoorbitocerebral mucormycosis; S/Sx, signs and symptoms; T2D, type 2 diabetes.

## DISCUSSION

Rhinoorbitocerebral mucormycosis is a rare and potentially lethal complication of COVID-19.^10^ Mortality rose to 43% in those with intracranial invasion, which was lower than the 90% reported by others.^2^ We presented 24 patients with mucormycosis associated with COVID-19 and identified the main visual signs on CT and MRI that radiologists should look for. Whenever indicated, we combined CT and MRI as compared to other studies in which only one imaging modality was used.^6^–^8^ Despite the better soft-tissue resolution of MRI, CT was better able to identify bone destruction, sequestration, and skull-base changes.

The mortality rate among our patients was 21%. Improved survival in our patients could be attributed partly to more effective treatment decisions based on improved diagnostics.

Several CT and MRI patterns were identified in our ROCM patients, discussed below, together with other possible findings noted in the literature.

### Sinonasal Involvement

**Figure 3:**
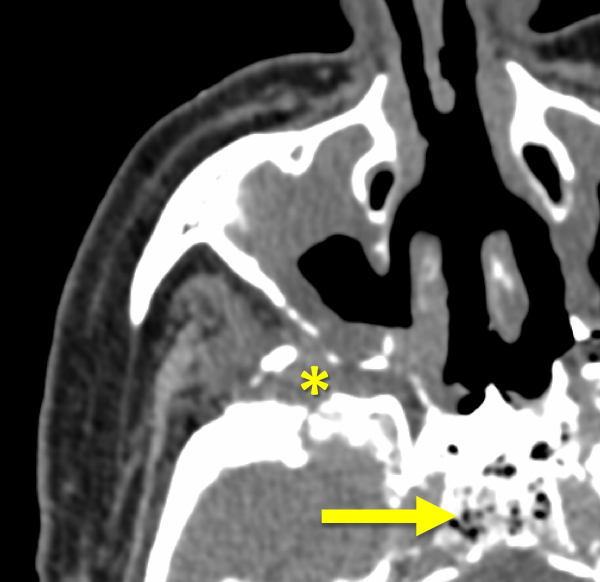
Computed Tomography of Paranasal Sinuses (Axial Plane, Soft-tissue Window). There is content in the right maxillar sinus with periantral invasion (asterisk). Air bubbles can be seen in the sphenoid bone (arrow).

The paranasal sinuses are air-filled structures; in the presence of ROCM they become filled with soft tissue-like content. Multiple sinus lesions were observed in about half of our patients.^11^ The paranasal sinus mucous membranes were thickened, with evidence of inflammatory changes. Soft tissue-like content filling the sinuses was associated with spreading to the surrounding tissues (Figure 3).

### Bone Wall Destruction

**Figure 4:**
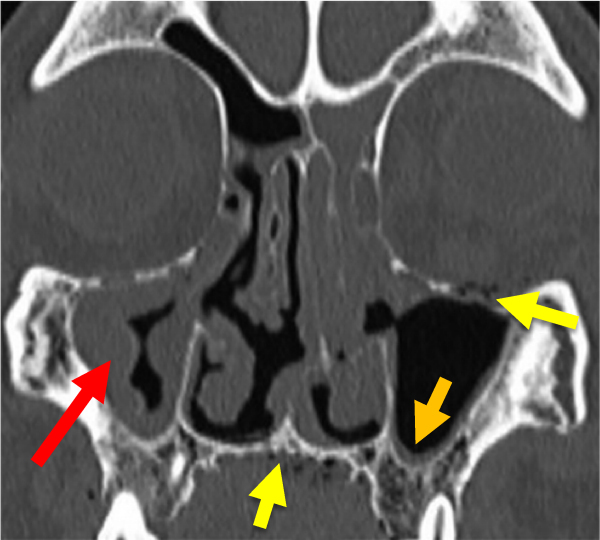
Computed Tomography of the Paranasal Sinuses (Coronal Plane, Bone Window). Polyps (red arrow) and mucosal thickening (orange arrow) can be seen in the right maxillar sinus and left ethmoid sinus. Intra- and extraosseous air bubbles (yellow arrows) indicate bone invasion.

In our patients, the spread of ROCM led to destruction of the bony walls enveloping the paranasal sinuses and orbits, as well as the base of the skull and the hard palate in 8 patients. Computed tomography revealed the presence of scattered intraosseous air bubbles, which may be specific to ROCM (Figure 4). This sign helped us to differentiate between ROCM destruction and other types of osteomyelitis. Whether intraosseous air bubbles are indeed characteristic of ROCM needs further verification.^12^

### Sequestration

**Figure 5:**
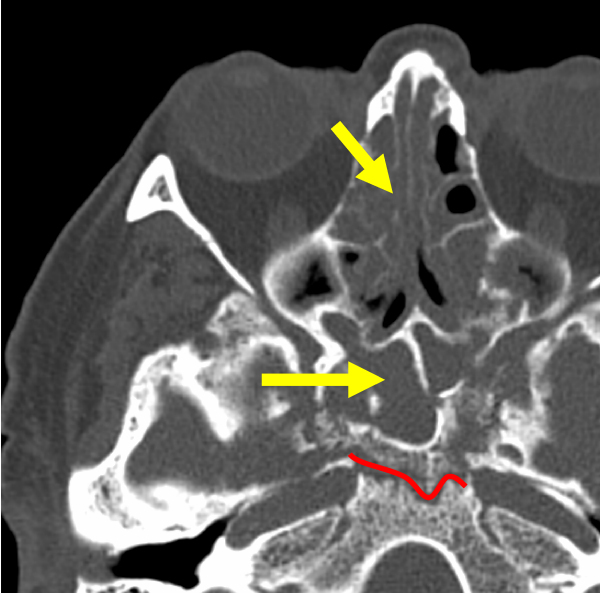
Computed Tomography of the Paranasal Sinuses, Axial Plane, and Bone Window. There is massive content in the ethmoid and sphenoid sinuses (arrows) with a line of sequestration in sphenoid bone (red line).

The symptoms of ROCM may lead patients to seek an initial consultation with a maxillofacial surgeon, as was the case with most of our patients. Upon initial examination, a fungal pathogen may be suspected. The problem with ROCM is that further bony destruction leads to sequestration, resulting in CT images similar to those seen in osteomyelitis. In our study, sequestration was found in 16 patients (Figure 5).

### Skull Base Involvement

Osteomyelitis of the skull base is a rare complication that usually occurs at the advanced stages of ROCM. The angioinvasive fungal process leads to a wide spread of infection into deep soft tissues through perivascular channels resulting in massive sequestration,^13^ which was noted in 6 of our patients (Figure 5).

### Orbital Involvement

**Figure 6:**
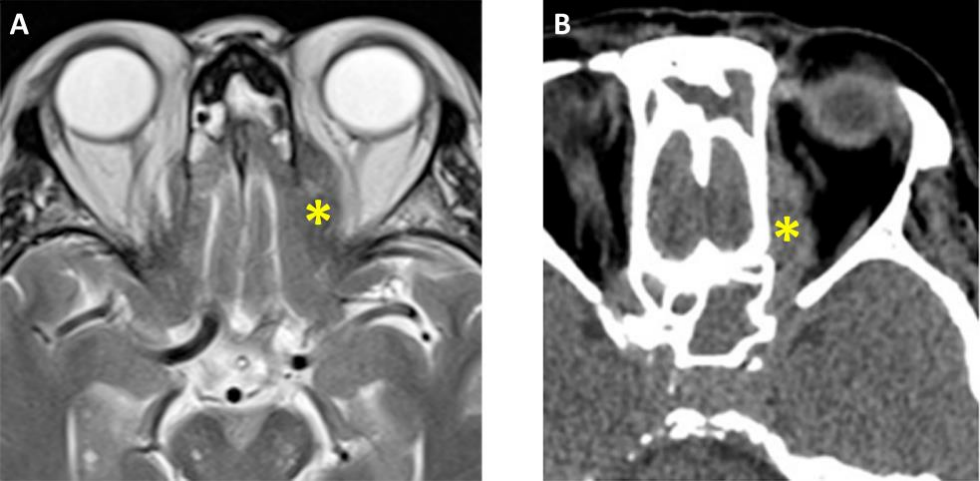
Magnetic Resonance Imaging and Computed Tomography of the Paranasal Sinuses (Axial Plane, Soft-tissue Window). **A:** T2-weighted MRI. **B:** Computed tomography. Note the better visualization of the left orbit invasion (asterisks) through the medial wall by MRI **(A)**, as opposed to CT **(B)**.

In 18 of our 24 patients, fungal mass prolapse into one or both orbits through destroyed or thinned paranasal sinus walls was noted, especially of the ethmoidal air cells of the ethmoid labyrinth. Spread into pterygopalatine fossae was also typical. As previously published, early signs of orbital involvement include soft-tissue infiltration and swelling of retroorbital fat around the oculomotor muscles.^13^ In our patients, retroorbital fat infiltration was better assessed on fat-suppressed MRI T2-WI images. Since orbital invasion usually occurred through the medial wall, inflammatory infiltration or abscess formation was observed along the medial side of the orbit with lateral displacement and edema of the medial rectus muscle. Noteworthy is the clearer visualization of the orbital component in MRI compared to CT scans (Figure 6).

### Optic Nerve Involvement

In cases of oculomotor muscles and optic nerve involvement, the main symptoms in our patients were periorbital edema, proptosis, decreased vision, and blindness. However, some patients suffered from eye muscle paralysis or weakness (ophthalmoplegia). Diffusion-weighted images revealed optic nerve infarction (*n*=4). In addition, physicians should be aware that the fungal invasion may involve the pia mater, which can be visualized on MRI as a post-contrast enhancement.^14^

### Diffuse Orbital Infiltration

Computed tomography did not reveal the extent of periorbital disease in patients suffering from severe exophthalmos and limited ocular motility. However, in 18 patients, there was clinical suspicion of diffuse orbital infiltration. Indeed, changes were visible in the 5 of these patients undergoing MRI: edema of the interconal tissue with dotted hypointense areas in T2-WI and FLAIR modes with areas of diffusion restriction and invasion or ischemic changes of the optic nerve; our findings were in line with the literature.^6^

### Orbital Apex Involvement

While not found in any of our patients, orbital apex involvement is clearly evident on MRI by an increased signal from the soft tissues of retrobulbar fat.^15^ Both the optic canal and superior orbital fissure may be involved. The latter is manifested clinically by the superior orbital fissure syndrome (SOFS), which is a complex of impaired function of the cranial nerves (III, IV, V, and VI) that enter the orbit through the superior orbital fissure (SOF). Clinical symptoms include: loss of sensation over the forehead, edema of the periorbital region, proptosis, pupil dilation, ptosis, ophthalmoplegia, loss of corneal reflex, loss of direct light reflex, presence of consensual reflex, loss of accommodation reflex, and lacrimal hyposecretion.^16^ The fungal infection (i.e. ROCM) can extend from the orbital apex posteriorly into the cavernous sinus and through the pterygopalatine fossa into the infratemporal fossa.^17^,^18^

### Intracranial Spread of Mucormycosis

**Figure 7:**
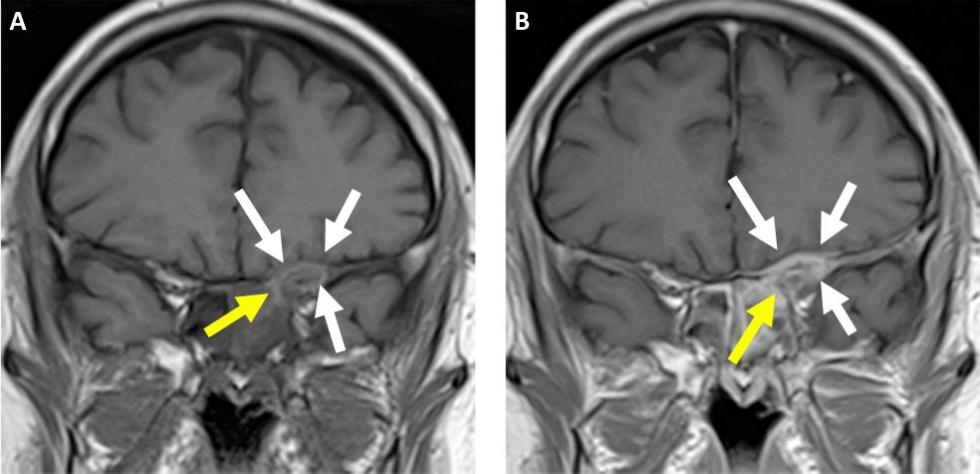
Magnetic Resonance T1-weighted Image of the Coronal Plane. **A:** Pre-contrast; **B:** Post-contrast. Note the spread of content from the sphenoid sinus (yellow arrow) to the dura mater (white arrows).

Intracranial spread of mucormycosis is usually by direct invasion through the lamina cribrosa, ethmoid walls, and frontal sinuses. Cranial fossae from the pterygopalatine fossa and along the internal carotid artery may also be directly infiltrated by mucormycosis, leading to occlusion or thrombosis of the central retinal artery, ophthalmic artery, or direct optic nerve infiltration.^19^ Perineural spread from the cavernous sinus along the trigeminal nerve can lead to predominant involvement of the posterior cranial fossa. An early intracranial spread is better assessed on contrast-enhanced MRI T1-WI images, manifested by leptomeningeal enhancement (Figure 7).

### Other Intracranial Manifestations

**Figure 8:**
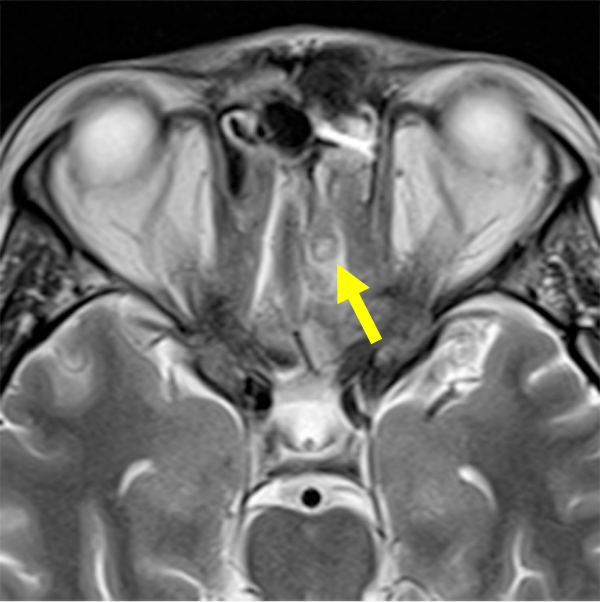
Magnetic Resonance T2-weighted Image of the Brain (Axial Plane). Note the ring-like abscess with perifocal edema (arrow).

Other intracranial manifestations of ROCM include infarcts and abscesses (Figure 8), the latter of which were noted in 3 of our patients. Fungal invasion of brain parenchyma manifests with fuzzy areas of MRI T2-WI hyperintensity and extravascular distribution. There is minimal perifocal edema and variable peripheral enhancement. Abscess formation is indicated by the development of a well-circumscribed mass with a liquefied central hyperintense MRI T2-WI nucleus showing diffusion restriction. Due to the poor immunogenic response of immunocompromised patients, ROCM abscesses may not show the typical well-defined margin enhancement as seen in the bacterial abscesses of non-immunodeficient patients.^20^

Middlebrooks et al.^7^ described a seven-variable CT-based diagnostic model for predicting acute invasive fungal sinusitis. The presence of any of seven patterns (spread to surrounding fat, orbits, pterygopalatine fossa, sphenopalatine foramen, nasolacrimal duct, lacrimal sac, bony destruction) provided 95% sensitivity and 86% specificity for fungal etiology. The presence of any two signs gave 88% sensitivity, 100% specificity, and 100% positive predictive value for a diagnosis of invasive fungal sinusitis.

### Differential Diagnosis Imaging Features

**Figure 9:**
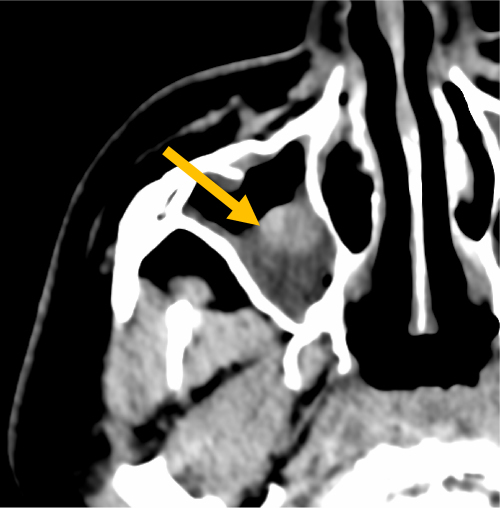
Computed Tomography of the Paranasal Sinuses (Axial Plane, Soft-tissue Window). Heterogeneous content with hyperdense areas is seen in the right maxillary sinus (arrow). Intraoperative data for this patient revealed a polyp and no evidence of a fungal process. Hence, hyperdense inclusions in the structure, which are considered to be pathognomonic for a fungal character, may indicate an unchanged polyp due to its high protein content.

Typical CT findings can usually differentiate between an invasive fungal process and chronic osteomyelitis of the skull and facial bones. However, diagnosing the difference between the two processes is challenging. For example, polyposis of the paranasal sinuses may represent the infectious process itself; however, it can also be a secondary reaction of the mucosa to the ROCM invasion of the adjacent bone. The diagnostic challenge is further complicated by the presence of typical polyps. Computed tomography was not so effective in differentiating between reactive polyps, mucormycosis, and normal polyps, since the classical sign of polyps—hyperdense inclusions—can be caused by all three processes^21^ (Figure 9) as was the case in 18 of our patients; this was better visualized by MRI.

## CONCLUSION

Our findings indicate that the degree of aggressiveness of ROCM may be determined using CT or MRI to identify the patchy bony destruction with scattered intraosseous air bubbles, infiltration, and invasion into the surrounding tissues. Mucosal thickening and polyposis are non-specific, so special attention should be paid to assessing surrounding tissue, such as the orbit, hard palate, maxilla, and brain. Computed tomography of the paranasal sinuses is the method of choice for suspected fungal infections such as ROCM. However, due to its high soft-tissue resolution, MRI is recommended if invasion beyond the sinuses is suspected. Although cavernous sinus involvement is reportedly common in ROCM,^11^ it was not identified in our patients.

Based on the analysis of this and other studies, several recommendations can be formulated for ROCM visualization:

1. The recommended CT brain scan protocol must capture the paranasal sinuses, as opposed to the routine sinus protocol characterized by low mA values on the X-ray tube and low soft-tissue resolution that cannot visualize intracranial invasion.
2. The routine MRI protocol should be extended by T2-sequence with fat suppression or STIR for periantral fat and muscle evaluation; for post-contrast MRI, a fat-suppressed 3D gradient echo (GRE) sequence should be used to achieve an accurate assessment of retrobulbar involvement and optic nerve damage.
3. Evaluation of the extent of ROCM involvement in bone and soft tissue may require both CT scans and MRI.

## Abbreviations

COVID-19: coronavirus disease 2019
CT: computed tomography
MRI: magnetic resonance imaging
ROCM: rhinoorbitocerebral mucormycosis
T2D: type 2 diabetes.
